# Impact of using peer role-playing on the clinical skills performance of pediatric trainees

**DOI:** 10.1186/s12909-023-04554-0

**Published:** 2023-09-08

**Authors:** Lingling Xu, Wangkai Liu, Xiaoyun Jiang, Yijuan Li

**Affiliations:** Department of pediatrics, The First Affiliated Hospital of Sun Yat-sen University, Guangzhou, 510080 China

**Keywords:** Role-playing, Pediatrics, Medical traineeship

## Abstract

**Objective:**

The aim of this study was to evaluate the impact of peer role-playing on the clinical skills performance of pediatric trainees.

**Methods:**

Seventy-eight clinical medicine trainees were randomly divided into a role-playing group and a traditional teaching group, with 39 students in each group. The role-playing group alternated between the roles of clinicians and patients, while the traditional teaching group received the bedside teaching mode of verbal instruction. After two weeks traineeship, mini-Clinical Evaluation Exercise(Mini-CEX) was used to evaluate the trainees’ competence in physician-patient communication and clinical practice. A questionnaire was given to the role-playing group to assess their satisfaction with the method.

**Results:**

The Mini-CEX scores showed that the role-playing group had superior clinical skills (p < 0.05), including communication, history taking, professionalism, organization, clinical skills, and physical examination, compared to the traditional teaching group. Furthermore, trainee satisfaction was high with the role-playing method,and the satisfaction were more than 95%.

**Conclusion:**

The role-playing method effectively improved the clinical skills of pediatric trainees, developed clinical communication skills, and enhanced the application of medical knowledge in a simulated medical environment.

## Introduction

Clinical traineeship is an important stage in medical education [1]. It plays a critical role in the clinical practice and role transformation of medical traineeship. Pediatrics is a “dumb department”, pediatric patients’ condition changes rapidly, anxiety and irritability frequently occur in their parents [2, 3]. At present, due to the tense doctor-patient relationship, medical students’ initiative in learning pediatric medicine is inadequate, and traditional clinical assessment practices are challenging to implement, becoming a bottleneck in the quality of clinical practice teaching in pediatrics.

Peer role-play has been shown to be an effective and popular model for teaching communication skills, especially in practicing history taking and doctor-patient communication [4, 5]. This approach allows learners to practice newly acquired skills in a low-risk environment without endangering actual patients and their families [5]. It is a valuable tool for consolidating pre-existing medical theoretical knowledge and applying it in practice settings [6]. The role-playing teaching method has not been widely used in pediatric clinical traineeship, and there is limited research in this area.

To address this, the study used Mini-Clinical Evaluation Exercise (Mini-CEX) to assess the clinical core competencies of medical students during their pediatric clinical traineeships, and to evaluate the effectiveness of role-playing exercises [7].

This study aims tocompare two teaching methods (role-playing and traditional) for clinical traineeship in pediatrics using the Mini-CEX to evaluate students’ clinical interviewing and doctor-patient communication skills.

## Objectives and methods

### Objectives

The present study aimed to investigate the efficacy of peer-role play training in improving the clinical skills of 78 clinical medical interns enrolled in a 5-year program at Sun Yat-sen University in 2021. The participants, consisting of 43 male and 35 female students, were divided into into four batches based on their practicum time. Each batch consisted of 3–4 students randomly assigned to a peer role-playing or traditional teaching group. The traineeship period for each group lasted for two weeks in pediatric practice.

### Methods

The study employed a controlled design. The peer role-playing group, composed of 39 students (male-to-female ratio = 7:6), engaged in six alternating sessions of simulated patient consultations, assuming the roles of parents and doctors. The typical cases involved in the two teaching groups include deficiency anemia, diarrheal disease, neonatal hyperbilirubinemia, nephrotic syndrome, bronchopneumonia, and septic meningitis.The traditional teaching group, consisting of 39 students (male-to-female ratio = 22:17), followed the traditional teaching approach, with the instructors demonstrating the medical history inquiry and physical examination techniques beside the patient’s bed. The patients were infants and toddlers, and the medical history was provided by their parents or caregivers. The two groups were not significantly different in terms of gender, age, and pre-practicum clinical exam scores (p > 0.05). Both groups received the same teaching resources, including the textbook, instructors, course hours, and testing difficulty. The chosen textbook was the ninth edition of the national higher education textbook “Pediatrics”, published by the People’s Health Publishing House.

### Teaching implementation

#### Traditional teaching group

Before the practicum period, the instructors selected common pediatric diseases and reviewed the latest developments in the relevant areas, preparing multimedia courseware to present the clinical features and diagnosis and treatment points of each disease. During the practicum period, the teaching process consisted of three steps. Firstly, the instructor introduced the clinical case and the diagnostic process, while demonstrating the medical history inquiry and physical examination. Secondly, the students in each group worked independently on interviewing the patient’s parents and examining the patient, while the instructor observed their performance. Pediatric clinical training uses infant and young child patients, and their medical history was obtained from their parents or caregivers. Finally, the instructor provided feedback on the students’ performance and addressed any questions raised by the students.

#### they adopted peer role-playing teaching method.

Prior to the traineeship, the teachers in the group prepared teaching plans for common and typical diseases of pediatric patients (all cases involving infants and toddlers), which were turned into typical case records. During the traineeship mentoring process,the following steps were followed: (1) The teacher introduced the case and diagnosis process at the bedside, and demonstrated medical history taking and physical examination. (2) In the classroom, the teacher taught the theoretical knowledge of role-playing to the students. Two mentoring teachers (chief pediatrician or above) demonstrated the role-playing of the patient’s family and doctor according to the script of typical cases. (3) Peer role-playing: Each group consisted of two students who acted as doctors to inquire about medical history (one student mainly asked questions, while the other student supplemented the conversation). (4) One to two students acted as the patient’s parents to present the medical history, and the parents orally informed the doctors about the patient’s physical examination. (5) Roles were randomly exchanged among the sudents. (6) Each typical case was practiced for 45 minutes. After the role-playing interviews, the teacher and medical students discussed the preliminary diagnosis, differential diagnosis, and treatment plan for the disease. Finally, the trainees provided feedback on their strengths and weaknesses during role-playing, and the teacher supplemented the strengths and weaknesses of the students during the interview and communication skills, and pointed out the techniques for collecting medical history and communicating.

### Evaluation methods

(1) At the end of the traineeship, both groups of students took a closed-book exam in pediatric theoretical knowledge. At the same time, Mini-CEX was used for assessment. The assessment content involed interviewing a real pediatric patient’s parent and conducting a physical examination on the patient under the guidance of the teacher. The two groups of patients consisted of infants and toddlers, and the medical history was recounted by the parents. Mini-CEX evaluates the pediatric five-year medical students in terms of medical history collection, physical examination, professional qualities, clinical judgment, doctor-patient communication, organizational efficiency, and overall ability by using a 7-item scale. Scoring is based on a 3-level, 9-point scoring system. Scores of 1–3 indicate below expectation, 4–6 indicate meeting expectation, and 7–9 indicate exceeding expectation. Mini-CEX evaluations are conducted by the same assessor.

(2) A questionnaire survey was conducted at the end of the role-playing group traineeship to evaluate the effectiveness of the role-playing teaching method. A self-evaluation scale was developed in the questionnaire survey with a Cronbach’s Alpha coefficient of0.805, indicating reliable scale consistency and reliability. The evaluation items included easier transition to real clinical cases, stimulating active learning interest, learning communication skills, empathy for parents, more profound memory of clinical characteristics, realistic imitation of clinical situations, and help in achieving learning goals. Ratings were divided into four categories: (1) very satisfied, (2) satisfied, (3) neutral, (4) dissatisfied.

### Statistical analysis

R software and Empower Stats statistical software were used for data analysis. Descriptive statistics for metric data are represented by $\overline x$ ± s, and t-tests are used to compare the role-playing group and the traditional teaching group. Count data was represented by frequency and percentage “n(%),“ and chi-square tests were used. P < 0.05 indicated statistical significance.

## Results

### Theoretical knowledge exam scores for both groups of trainees

The score for the role-playing group on the theoretical knowledge exam was 92.9 ± 4.7 points and 92.5 ± 5.1 points for the traditional teaching group. There was no statistically significant difference in the scores between the two groups (P = 0.580).

### Mini-CEX evaluation results for both groups of trainees

**Figure 1:**
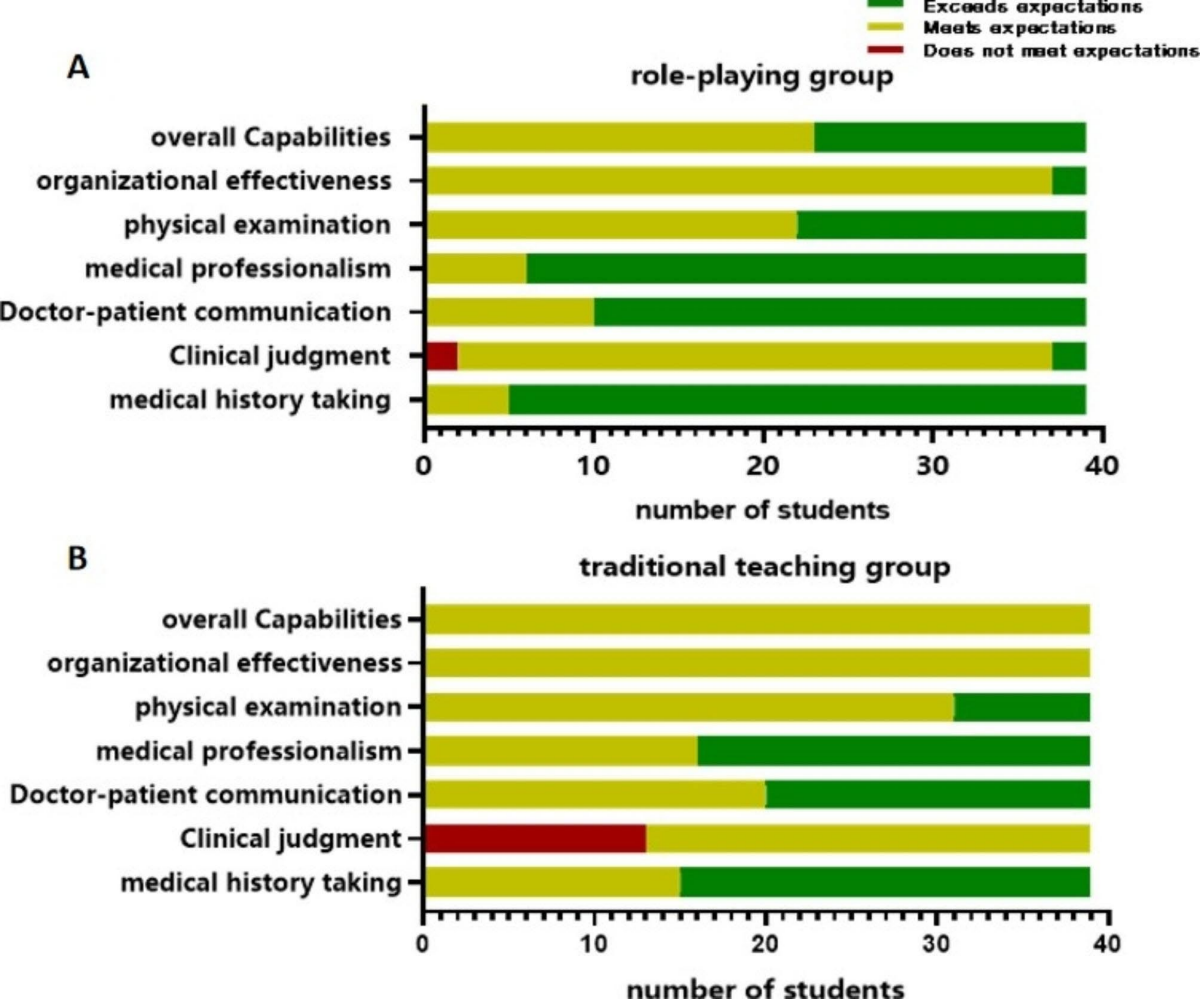
The scale scores of Mini-CEX assessment between the two groups **1A:** role-playing group; **1B:** traditional teaching group

All students completed the assessment within (37.0 ± 0.3) minutes. Each student received a complete assessment, and all assessors provided feedback to the students on-site, with an average feedback time of (5.9 ± 0.4) minutes. The Mini-CEX evaluation results showed that the role-playing group performed better than the traditional teaching group in aspects such as medical history collection, clinical judgment, doctor-patient communication, professional qualities, physical examination, and overall clinical skills.The P value was less than 0.05 for all aspects (Table 1). The level scores of the Mini-CEX rating scale for the role-playing group and the traditional teaching group are shown in Figure1.

**Table 1:** The scale scores of Mini-CEX assessment between the two groups

Itiems	Role-playing group, cases(%)	Traditional teaching group, cases(%)	P-value
Total cases	39(100%)	39(100%)	
Medical history taking			0.010
Meets expectation	5 (12.8%)	15 (38.5%)	
Exceeds expectation	34 (87.2%)	24 (61.5%)	
Disease Determination			0.003
Below expectation	2 (5.1%)	13 (33.3%)	
Meets expectation	35 (89.7%)	26 (66.7%)	
Exceeds expectation	2 (5.1%)	0 (0.0%)	
Doctor-patient communication			0.020
Meets expectation	10 (25.6%)	20 (51.3%)	
Exceeds expectation	29 (74.4%)	19 (48.7%)	
Professionalism			0.012
Meets expectation	6 (15.4%)	16 (41.0%)	
Exceeds expectation	33 (84.6%)	23 (59.0%)	
Physical Examination			0.029
Meets expectation	22 (56.4%)	31 (79.5%)	
Exceeds expectation	17 (43.6%)	8 (20.5%)	
Organizational effectiveness			0.152
Meets expectation	37 (94.9%)	39 (100.0%)	
Exceeds expectation	2 (5.1%)	0 (0.0%)	
Overall Capabilities			< 0.001
Meets expectation	23 (59.0%)	39 (100.0%)	
Exceeds expectation	16 (41.0%)	0 (0.0%)	

The scale scores of Mini-CEX assessment between the role-playing group and traditional teaching group were shown in Figure 1.

### Satisfaction survey results of trainees in the role-playing group

According to the feedback from the students, they were all very satisfied with the role-playing teaching method, and there were no dissatisfied students (Table 2).

**Table 2:** Student satisfaction evaluation of the role-playing group

Items	Very satisfied(n)	Satisfied (n)	Neutral (n)	Dissatisfied (n)	Satisfaction(%)
easier transition to real clinical scenarios	38	1	0	0	100
stimulating an interest	38	1	0	0	100
improving communication skills	38	1	0	0	100
understanding parents’ feelings	36	2	1	0	97.4
deeper understanding of clinical skills and theoretical knowledge	37	1	1	0	97.4
realistic imitation of clinical situations	38	0	1	0	97.4
helping to achieve learning objectives	38	1	0	0	100

## Discussion

The study indicates that both standardized patients and peer role-playing are effective tools for communication skills training in the healthcare field, and are superior to traditional methods such as lectures and verbal instruction [8]. However, in terms of cost-effectiveness, peer role-playing has an advantage over standardized patient training, which requires payment for trained actors to simulate patients with medical or psychological conditions. Instead, role-playing involves trainees taking on different roles or characters themselves, making it a more affordable option in public hospitals and other healthcare settings. Studies have shown that role-playing method is a relatively new learning method [5, 9]. Role-play teaching method is widely used in medical education [10]. However, peer role-playing is rarely used in pediatric medical education, especially in pediatric trainee teaching.

Our study applied peer role-playing in the pediatric clinical teaching of undergraduate students.Our study confirmed that peer role-play was an effective clinical teaching method for pediatric trainees, consistent with Brittany’s findings [4]. Our research showed that trainees’ clinical skills, including history taking, clinical reasoning, doctor-patient communication, and professionalism, significantly improved using a role-playing method. This approach had advantages over traditional didactic teaching, such as enhancing empathy and confidence, promoting student initiative and participation, and facilitating reflection and understanding of knowledge. Using role-playing in pediatric medical education helped trainees develop non-technical skills, such as communication and teamwork, and could prevent conflicts during doctor-patient interactions.Incorporating mini-CEX as a formative assessment tool into apprentice teaching allowed for feedback to be given on student performance during clinical practice, ultimately facilitating the development of their clinical skills.

However, there are several limitations to this study that should be considered in future research, such as the small sample size and the single-center study design. Additionally, the impact of peer role-playing on subjective outcomes, such as trainees’ confidence, attitudes, and communication skills, needs further investigation.

In conclusion, the findings of our study suggest that peer role-playing is an effective method of teaching clinical skills to pediatric trainees. The method has the potential to be widely adopted in pediatric medical education as it promotes the development of important non-technical skills and enhances trainees’ clinical skills performance. Future research should aim to investigate the optimal conditions and protocols for the implementation of role-playing in medical education, and to explore its impact on long-term patient outcomes and trainee retention.

Based on the results of this study, we suggest that medical educators incorporate role-playing into their teaching methods to enhance clinical skills training. Additionally, medical institutions should provide opportunities for trainees to practice clinical skills in simulated and safe environments, as well as facilitate trainee engagement and feedback to improve the effectiveness of clinical skills training. Finally, further research is needed to evaluate the impact of role-playing on trainee confidence, attitudes, and communication skills, as well as its long-term impact on patient outcomes and trainee retention.

## Data Availability

All data sets generated for this study were included in the manuscript.
